# Resilience Building in Students: The Role of Academic Self-Efficacy

**DOI:** 10.3389/fpsyg.2015.01781

**Published:** 2015-11-27

**Authors:** Simon Cassidy

**Affiliations:** Psychology and Public Health, University of SalfordSalford, UK

**Keywords:** resilience, self-efficacy, adversity, student, learning

## Abstract

Self-efficacy relates to an individual's perception of their capabilities. It has a clear self-evaluative dimension leading to high or low perceived self-efficacy. Individual differences in perceived self-efficacy have been shown to be better predictors of performance than previous achievement or ability and seem particularly important when individuals face adversity. The study investigated the nature of the association between academic self-efficacy (ASE) and academic resilience. Undergraduate student participants (*N* = 435) were exposed to an adverse situation case vignette describing either personal or vicarious academic adversity. ASE was measured pre-exposure and academic resilience was measured post-exposure. ASE was correlated with, and a significant predictor of, academic resilience and students exhibited greater academic resilience when responding to vicarious adversity compared to personal adversity. Identifying constructs that are related to resilience and establishing the precise nature of how such constructs influence academic resilience will assist the development of interventions aimed at promoting resilience in students.

## Introduction

### Psychological resilience

A shift in emphasis in mental health policy to include promotion of positive mental health as a preventative measure (WHO, 2005), together with the identification of resilience and coping as one of eight positive mental health grouping (Parkinson, 2008), underlines the value of studies examining resilience. Abiola and Udofia (2011) suggest resilience is associated with increased quality of life, wellbeing and functional capacity in times of adversity. Although there is an intuitive appreciation for the “meaning” of resilience and what it infers (about the individual), consensus in defining psychological resilience, both conceptually and operationally as a measurable construct, has yet to be reached. As Friedland (2005) notes, perspectives on resilience are highly diverse and the concept of resilience is highly elusive. In an attempt to illustrate the concept, Gilligan (2001) uses the example of a resilient child as a child who does better than they ought to, bearing in mind what has happened to them. Friedland (2005) goes on to discuss resilience as inferring hardiness, toughness, and resistance, along with—somewhat paradoxically—elasticity and flexibility. This suggests that resilience is both multi-faceted and multi-leveled and the range of available definitions reflects this in both their depth and their breadth. Resilience is described by Hamill (2003) as competence in the face of adversity and by Gilligan (2001, p. 5) as “a set of qualities that help a person to withstand many of the negative effects of adversity.” Pooley and Cohen (2010, p. 34) define resilience as “the potential to exhibit resourcefulness by using internal and external resources in response to different contextual and developmental challenges….” Garmezy and Masten (1991, p. 459) refer to resilience as “the process of, capacity for, or outcome of successful adaptation despite challenging circumstances.” Abiola and Udofia (2011) offer a fuller account, discussing resilience in terms of inner strength, competence, optimism, flexibility, and the ability to cope effectively when faced with adversity, minimizing the impact of risk factors, such as stressful life events, and enhancing the protective factors, such as optimism, social support, and active coping, that increase people's ability to deal with life's challenges.

Although seemingly diverse, most definitions of resilience feature adaptive, resourceful and innovative enabling responses to adversity, threat or challenge as a core element. As such, resilience is considered an asset or strength, a desirable and advantageous quality, characteristic or process that is likely to impact positively on aspects of an individual's performance, achievement, health, and wellbeing (Bartley et al., 2010).

As is common with many psychological constructs—self-efficacy for example (Bandura, 1997)—, there is debate around the existence and relevance of a global resilience construct. Instead, there is a strong argument for resilience to be considered—and measured—as a context-specific construct. Riley and Masten (2005) explain the need to contextualize resilience on the basis that judgments about risk and adversity relate directly to events and contexts, as do evaluations of competencies and outcomes. Both Liddle (1994) and Waxman et al. (2003) refer to the need to contextualize resilience in order to generalize findings from resilience studies and in order to consider specific practical implications for building resilience. The present study examines resilience in the context of education and learning (i.e., *academic* resilience), considering resilience as an asset and seeking to identify factors that may contribute to resilience promoting interventions for students, suggested by Zautra (2009) to have long-term benefits.

### Academic resilience

Wang et al. (1994) refer to academic resilience as an increased likelihood of (academic) success despite environmental adversities. Resilient students are described by Alva (1991) as those who maintain high motivational achievement and performance even when faced with stressful events and conditions that place them at risk of poor performance and by Waxman et al. (2003) as those who succeed at school despite the presence of adverse conditions.

As is the case with general resilience, work focussing on academic resilience has led to the emergence of apparently distinct yet related concepts and constructs, each aiming to address a seemingly similar issue. Although drawing some explicit distinctions between their own constructs and resilience (Perkins-Gough, 2013), both Duckworth and Dweck provide significant contributions to the field of academic resilience with their work on “grit” and “mindset.” Duckworth describes grit as an individual's tendency to sustain interest, passion, effort and persistence toward achieving long-term future goals (despite challenges and failures) and reports grit as a better predictor of academic success than IQ (Duckworth et al., 2007; Duckworth, 2013) or talent (Duckworth and Quinn, 2009). Dweck's (2006, 2010) work on mindset has led to the identification of two types of mindset, fixed and growth. A fixed mindset describes individuals with fixed beliefs regarding their level of intelligence and ability, which they believe remain stable. A growth mindset instead describes individuals who view their intelligence and ability simply as a basis for development and believe that challenges, including failure, are opportunities to develop their capacity for success through effort and practice. The influence of mindset is emphasized further by Snipes et al. (2012), who consider a growth mindset to be a major contributory factor in the development of grit. Despite noted dissimilarities—Duckworth considers resilience to be only one factor explaining grit (Perkins-Gough, 2013)—there are clear overlaps between academic resilience and the constructs proposed by Duckworth and Dweck, and their relevance is illustrated by Farrington et al. (2012) who reports that the combination of a growth mindset and grit in students is been associated with higher academic grades.

Another construct, closely related to academic resilience, proposed by Martin and Marsh (2008, 2009) is academic buoyancy. Described as the “capacity to overcome setbacks, challenges, and difficulties that are part of everyday academic life.” (Martin, 2013, p. 488) it is seen as distinct from academic resilience, which instead relates to the capacity to overcome significant adversity that threatens a student's educational development. Martin (2013) does present evidence that whilst buoyancy and resilience are related, buoyancy better predicts low-level negative outcomes and resilience better predicts major negative outcomes, which aligns with Martin and Marsh's (2008) earlier description of buoyancy as reflecting “everyday” academic resilience.

Waxman et al. (2003) suggest that studying resilient students will provide important implications for improving the education of students at risk of academic failure and evidence already exists supporting the relevance of academic resilience. McLafferty et al. (2012) reported that both resilience and emotional intelligence predicted coping at university, with resilience as the only significant unique predictor of coping subscales for grades, attendance, and studying. Furthermore, Abiola and Udofia (2011) reported higher perceived stress, anxiety and depression in low resilience medical students following completion of a major professional examination.

Waxman et al. (2003) note that resiliency refers to factors and processes that limit negative behaviors associated with stress and result in adaptive outcomes in the presence of adversity. They discuss the value of resilience studies that identify differences between resilient and non-resilient students and that focus on alterable factors to design more effective educational interventions. They suggest that focusing on educational resilience and those factors that can be altered to promote resilience may help address the gap in achievement between those students who are successful and those who are at risk of failure. Like Wagnild (2009), Waxman et al. (2003) further suggest that rather than being fixed, academic resilience can be promoted by focussing on alterable factors including social competence, problem-solving skills, autonomy, a sense of purpose (Bernard, 1993), motivation and goal orientation, positive use of time, family life, and learning environment (McMillan and Reed, 1994). The potential for building resilience, together with Munro and Pooley's (2009) suggestion that resilience may mediate adversity and success in university students and Hamill's (2003) prioritizing of self-efficacy over other resilience factors, provides the major premise for the present study examining academic self-efficacy (ASE) as a factor influencing student responses to academic adversity.

### Resilience and self-efficacy

Waxman et al. (2003) proposes that academic resilience research needs to examine indicators of resiliency in order to identify what processes can promote protective mechanisms and calls for more affective and motivational training for students to assess their impact on students' affective and motivational outcomes. Aiming to provide a more “expansive” analysis of the factors related to academic resilience, Martin and Marsh (2006) reported self-efficacy, planning, persistence, anxiety, and uncertain control as predictors of academic resilience. Using class participation (behavioral) and enjoyment at school (cognitive-affective) as educational outcome constructs and general self-esteem (global-affective) as a psychological outcome construct, Martin and Marsh hypothesized that the outcome constructs were consequential to students' capacity to effectively deal with challenge, adversity and setbacks experienced in a school setting. As hypothesized, academic resilience was the strongest—relative to the other five motivational and engagement factors—predictor of each of the outcome measures. Analysis to determine students' profiles according to academic resilience revealed that resilient students were high in self-efficacy, persistence and planning and low in anxiety and uncertain control. Hamill (2003) also reported self-efficacy as an important characteristic that distinguished resilient and non-resilient 16–19 year old students.

The pursuit of those factors that distinguish resilient from non-resilient individuals and the promotion of resilience have been at the center of existing research in the field resilience (Hamill, 2003). There is sufficient evidence indicating that self-efficacy is one resilience factor worthy of further study in this respect. Self-efficacy emerged as a central facet in Albert Bandura's Social Cognitive Theory, where is it described as “the belief in one's capabilities to organize and execute the course of action required to manage prospective situations” (Bandura, 1995, p. 2). In educational studies, individual differences in perceived self-efficacy have often been shown to be better predictors of performance than either previous achievement or ability (Cassidy, 2012).

Like resilience, self-efficacy is context specific and seems particularly important when individuals face adversity, when positive self-efficacy beliefs are associated with increased motivation and perseverance (Bandura, 1997; Bandura et al., 2001) and an increased likelihood of rejecting negative thoughts regarding own capabilities (Ozer and Bandura, 1990).

Self-efficacy is considered to be the foundation of human agency (Bandura et al., 1999) and is referred to as an important protective factor regulating human functioning and emotional wellbeing through cognitive, motivational, affective, and selective processes (Hamill, 2003). And whilst Bandura (1993) does describe how self-efficacy operates to contribute toward academic development—stating that students' beliefs in their efficacy to regulate their own learning and master academic activities determine their aspirations, level of motivation and academic accomplishment—there is a lack evidence-based detail accounting for exactly what high self-efficacious individuals *do* that impacts positively on academic outcomes; as noted by Hamill (2003), despite an abundance of self-efficacy focussed research, relatively little work has examined how self-efficacy relates to resilient behaviors exhibited in response to adversity.

### Present study

Operationalizing academic resilience as students' cognitive-affective and behavioral responses to academic adversity, the present study seeks to establish examples of context-specific resilience factors and resilience responses to academic adversity. Self-efficacy has been identified as a key construct in previous studies examining factors affecting academic achievement (e.g., Cassidy, 2012), where high self-efficacy is commonly reported as associated with better academic performance. What has not been clearly established in these studies are the specific responses of self-efficacious students to instances of academic adversity, when self-efficacy beliefs are particularly relevant because of their association with increased motivation and perseverance (Bandura, 1997) and resistance to negative thought (Ozer and Bandura, 1990). Hamill (2003) has explored this issue but using generalized measures of self-efficacy and coping responses in the context of general stressful life events in a small sample of 16–19 year old students—limitations which Hamil partly acknowledges. Hamil reported an association between self-efficacy and resilience, adding support to the merits of the present study and its aim of uncovering differences in context-specific resilience responses adopted by self-efficacious and non-self-efficacious students, and the study's longer-term objective of promoting resilient responses in students.

Riley and Masten (2005, p. 13) define resilience as “referring to patterns of positive adaptation in the face of adversity…,” and describe resilience as requiring “that significant adversity or threat to adaptation or development has occurred” and “that functioning is okay, either because adequate adaptation was sustained over a period of adversity or because recovery to adequate functioning has been observed.” In order to represent the key constituents of resilience (i.e., adversity and positive adaption) in a context-specific and authentic manner to serve the purposes of the study, an academic adversity case vignette and a response to academic adversity scale (Academic Resilience Scale-30) were developed [see Section Academic Resilience Scale-30 (ARS-30)].

The content of the case vignette was intended to represent adversity in a context-specific academic setting that undergraduate students would consider authentic despite its hypothetical nature. The vignette describes academic failure and its wider impact as an example of authentic adversity for students. Although there is some debate in the existing literature on the specific effects of, and perceptions of, negative feedback (e.g., Kluger and DeNisi, 1996), reference in the vignette to failure and the wider negative impact of such failure was considered to be sufficient to instill academic adversity. There are two versions of the vignette presented in Section Academic Resilience Scale-30 (ARS-30), *personalized* and *vicarious*. The personalized vignette asks that participants imagine that they are personally facing adversity and how they would respond, whilst the vicarious vignette asks participants to imagine that a fellow student is facing adversity and how that student should respond. The vicarious vignette was developed in order to explore any differences between responses to personal adversity and responses advocated for a fellow student facing adversity, and to examine in what way self-efficacy beliefs are associated with any differences. Gaining such insight may be valuable for resilience building interventions, whereby any differences in personal and advocated responses can be used to highlight self-limiting responses or belief systems that may also limit students' capacity for acting in advocate roles, including peer-assisted learning programmes.

Based on previous studies it is anticipated that findings will reveal a positive relationship between ASE and academic resilience, although it is unclear which of the 30 responses to academic adversity will present as most pivotal in defining differences in academic resilience between lower and higher ASE students. Because self-efficacy is a “self” construct most closely related to personal functioning, it is anticipated that any association between self-efficacy and resilience will be more pronounced in responses to the personal adversity vignette as compared to the vicarious adversity vignette.

## Method

### Participants and design

The sample comprised 435 British undergraduate students (see Tables 1, 2). The study adopted a self-report questionnaire-based design with correlational and between-subjects components. Academic self-efficacy and academic resilience were measured during a single data collection point in participants' first, second, or third year as undergraduates. Gender, age, and year of study data were also collected. Whilst the gender bias evident within the sample was not desirable, that over 80% of the sample were female is representative of a typical student intake, at least in psychology (Bourne, 2014).

**Table 1:** **Total sample details**.

	**Total sample**	**Male**	**Female**	**Year 1**	**Year 2**	**Year 3**
*N*	435	76	357	326	45	63
Mean Age *(SD)*	22.6 (6.4)	22.9 (6.9)	22.6 (6.2)	22.5 (6.8)	22.6 (5.4)	23.4 (4.3)

**Table 2:** **Sample details by vignette group**.

**Vignette group**	***N*; Total**	**Mean Age *(SD)***	***n*; Males**	***n*; Females**	***n*; Year 1**	***n*; Year 2**	***n*; Year 3**
Personal	224	22.7 (6.8)	42	180	167	23	31
Vicarious	211	22.5 (5.8)	34	176	157	22	32

### Materials

#### General academic self-efficacy scale (GASE)

This is 23 item context-specific scale measuring student ASE. The General Academic Self-Efficacy Scale was adapted from the Health Student Self-Efficacy (HSSE) Scale originally developed by Eachus (1993) as a measure of self-efficacy beliefs in students on health-related courses involving clinical training and practice. Cassidy and Eachus (2002) revised the HSSE scale, removing reference to clinical placements, and developed the GASE scale for use with general undergraduate student populations. Participants record their level of agreement with each of the 23 items along a 9-point Likert scale from strongly agree to strongly disagree. The scale contains both positively and negatively worded items, examples of which include “I know I have the ability to complete this course successfully” and “I have some doubts about my ability to grasp some of the topics taught on this course.” Scores for negatively worded items are reversed so that a high GASE score indicates high (or positive) ASE. Scores for the 23 items are summed providing a total scale score between 23 and 207. Cassidy and Eachus (2002) report high internal (α = 0.86) and external (*r* = 0.71) reliability for the GASE scale and construct validity is further demonstrated through significant correlations with academic locus of control and computer user self-efficacy. A similarly high alpha (α = 0.84, *N* = 434) is reported in the present study.

#### Academic resilience scale-30 (ARS-30)

In the absence of a suitable measure of academic resilience, the ARS-30 was developed as a context-specific measure of student response to academic adversity. Scale items represent a sample of relevant positively and negatively phrased behavioral and cognitive-affective responses that participants have to rate as likely or unlikely along a 5-point Likert scale following exposure to the personal or vicarious adversity case vignette:

*Personal Vignette:* You have received your mark for a recent assignment and it is a “fail.” The marks for two other recent assignments were also poorer than you would want as you are aiming to get as good a degree as you can because you have clear career goals in mind and don't want to disappoint your family. The feedback from the tutor for the assignment is quite critical, including reference to “lack of understanding” and “poor writing and expression,” but it also includes ways that the work could be improved. Similar comments were made by the tutors who marked your other two assignments.
*Vicarious Vignette:* John has received a mark for a recent assignment and it is a “fail.” The marks John received for two other recent assignments were also poorer than he would want as he is aiming to get as good a degree as he can because he has clear career goals in mind and doesn't want to disappoint his family. The feedback John received from the tutor for the failed assignment is quite critical, including reference to “lack of understanding” and “poor writing and expression,” but it also includes ways that the work could be improved. Similar comments were made by the tutors who marked John's other two assignments.

Scoring of positively phrased items was reversed so that a high ARS-30 score indicated greater academic resilience. Cronbach's alpha for the combined (α = 0.89, *N* = 432), personalized (α = 0.88, *n* = 224) and vicarious vignette (α = 0.85, *n* = 208) all reached acceptable levels indicating internal reliability and construct validity (Nunnally and Bernstein, 1994). Analysis of the relationships between ARS-30 scores and ASE and differences between personal and vicarious responses to adversity further supported the construct validity of the ARS-30 as a measure of academic resilience (see Section Results).

Exploratory factor analysis [principle component with oblique (promax) rotation] was conducted to explore the structure of the ARS-30. Sampling adequacy was verified (KMO = 0.91) and whilst initial analysis revealed seven factors with eigenvalues of 1.0 or above (Kaiser, 1960) explaining 55.75% of the variance, the scree plot inflection (Cattell, 1966) supported retention of only three factors (Hatcher, 1994; Stevens, 2002). The three factor model explained 40% of the variance with all items—except one, which loaded at 0.29—loading above 0.3 (Field, 2014). Interpretation of Item-factor clustering suggests that factor 1 represents positive or adaptive responses to adversity, factor 2 represents negative or non-adaptive responses to adversity and factor 3 represents long-term future aspirations. Thus, factors 1 and 2 may simply represent two aspects of the same underlying generalized academic resilience construct. This is partly supported by Schmitt and Stults (1985) and Spector et al. (1997) who report that reverse-phrased items commonly load on different factors, even in the absence of multiple constructs, and by the inter-factor correlation (−0.45) between factors 1 and 2. That factor 3 aligns with closely associated and relevant constructs such as Duckworth's “grit,” which has its basis in long-term goals, suggests that a three factors solution presents an interpretable solution to the ASR-30.

## Procedure

The study was carried out in accordance with the recommendations of the British Psychological Society Code of Ethics and Conduct and the Research, Innovation and Academic Engagement Ethical Approval Panel, University of Salford with written informed consent from all subjects in accordance with the Declaration of Helsinki.

After completing the GASE scale, participants were randomly assigned to one of the adversity vignette conditions and completed the ARS-30 (personal or vicarious). Data collection was anonymous to improve the validity of responses. A median-split approach was used to create discrete groups according to scores on the GASE. Participants with scores equal to or below the GASE sample median of 148 were assigned to the lower ASE group, while participants scoring above the median were assigned to the higher ASE group. Whilst the median-split approach is criticized on the basis of loss of statistical power and the potential for spurious outcomes in cases of multiple variables (MacCallum et al., 2002; Irwin and McClelland, 2003), the approach has received support in terms of producing meaningful findings that are understood by, and accessible to, a wider audience where statistical power and effect are not necessarily reduced (Farrington and Loeber, 2000). Thus, the use of dichotomization here is defended on the basis that correlational and regression analysis were also performed for the main analysis using GASE scores as a continuous variable; that the mean difference between groups (30.3) provided, it is suggested, sufficient numerical distance between groups; and the wish to illustrate, in a meaningful way, distinctions between groups in terms of specific responses to adversity.

## Results

Significant positive correlations between ASE and academic resilience were observed for the combined vignette groups (medium effect size *r* = 0.34, Cohen, 1988) and for the personal (large effect size *r* = 0.51) and vicarious vignette groups (small effect size *r* = 0.21) separately. Academic self-efficacy was a significant predictor of academic resilience explaining 26.2% of variance in resilience in the personal vignette group, 4.6% in the vicarious vignette group, and 14% in the combined groups (see Table 3).

**Table 3:** **Zero order correlations and regression analysis with academic self-efficacy (ASE) as a predictor of academic resilience**.

		**Academic resilience (combined groups)**	**Academic resilience (personal group)**	**Academic resilience (vicarious group)**
Zero order correlations	Academic self-efficacy	0.34^**^	0.51^**^	0.21^*^
		(*N* = 431)	(*n* = 224)	(*n* = 207)
Model statistics	*F* (*df*)	55.45 (1,429)	78.83 (1,222)	9.78 (1,205)
	*P*	<0.001	<0.001	=0.002
	*R*^2^	0.14	0.262	0.046
Predictor statistics	β	0.25	0.37	0.13
	*T*	7.45	8.88	3.13
	*P*	<0.001	<0.001	=0.002

* *p = 0.002*,

** *p < 0.001*.

A 2(vignette: personal vs. vicarious) × 2(ASE: lower vs. higher) between-subjects factorial ANOVA was conducted to examine differences in academic resilience between personal and vicarious vignette groups as a function of ASE (see Table 4).

**Table 4:** **Mean academic resilience scores by vignette group and academic self-efficacy (ASE) Group**.

**Vignette group**	**Mean (*****SD*****) academic resilience**			**Effect size *d***
	**Lower ASE**	**Higher ASE**	**Total**	
Personal	110.96	122.14	116.25	0.86
	(13)	(12.87)	(14.07)	
	*n* = 118	*n* = 106	*N* = 224	
Vicarious	126.75	130.15	128.51	0.30
	(13.05)	(9.54)	(11.47)	
	*n* = 100	*n* = 107	*N* = 207	
Total	118.20	126.16	–	0.58
	(15.20)	(11.99)		
	*N* = 218	*N* = 213		
Effect Size *d*	1.21	0.71	0.96	(2 × 2) 0.33

**Figure 1:**
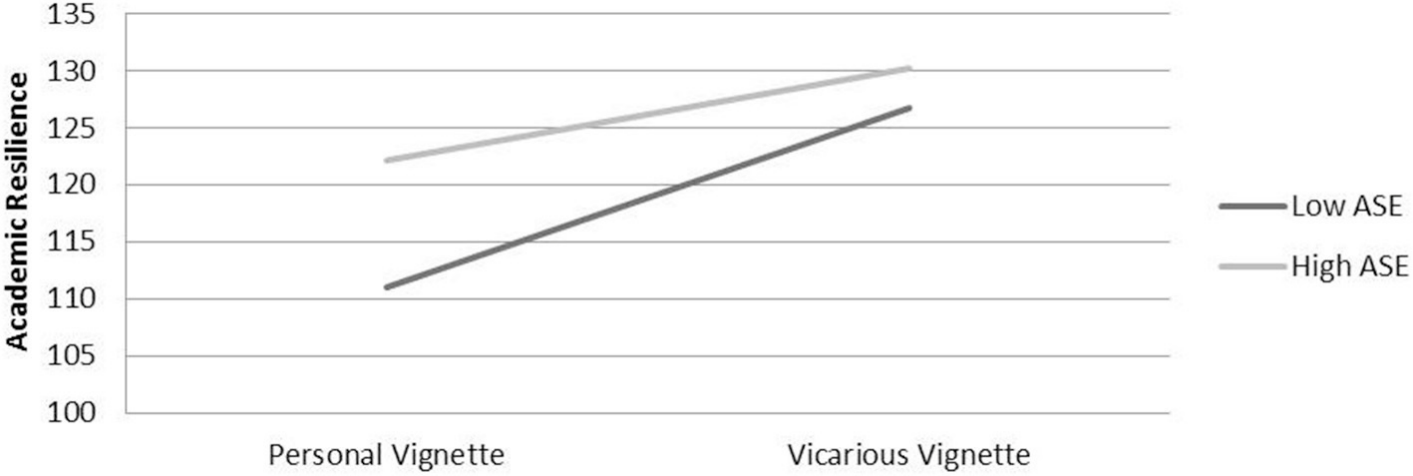
**Mean academic resilience by ASE group vignette type**.

There were significant main effects for vignette group [*F*_(1, 427)_ = 101.91, *p* < 0.001, *d* = 0.96], such that the vicarious vignette group reported significantly higher academic resilience (*M* = 128.51, *SD* = 11.47) than the personal vignette group (*M* = 116.25, *SD* = 14.07), and for ASE group [*F*_(1, 427)_ = 38.26, *p* < 0.001, *d* = 0.58], with the higher ASE group reporting significantly higher academic resilience (*M* = 126.16, *SD* = 11.99) than the lower ASE group (*M* = 118.20, *SD* = 15.20). A significant interaction effect [*F*_(2, 427)_ = 10.9, *p* < 0.001, *d* = 0.33] indicated that the influence of ASE on increasing academic resilience was significantly greater in the personal vignette group, where the effect size was large (*d* = 0.86), than in the vicarious vignette group, where the effect size was small (*d* = 0.30) (see Figure 1).

Both lower and higher ASE groups scored higher academic resilience when responding to the vicarious vignette than when responding to the personal vignette, though the effect size was larger for the lower ASE group (large ES *d* = 1.21) than for the higher ASE group (medium ES *d* = 0.71).

Table 5 shows ASR-30 (personal vignette) mean item scores by ASE group (lower and higher). A One-way MANOVA was performed on these data with ASE group (lower vs. higher) as the independent variable and ASR-30 item scores as the dependent variables. There was a significant multivariate effect [*F*_(1, 222)_ = 2.971, *p* < 0.001] and significant univariate effects. Significant univariate effects are denoted by “^*^”and reflect scores indicating significantly higher academic resilience for the higher ASE group on all items except items 1, 6, 14, 26, and 29, where any differences were non-significant (*p* > 0.05). Effect sizes were medium (*d* ≥ 0.5) for 12 of the items and small (*d* ≥ 0.2 < 0.05) for the remaining 13 items where a significant group difference was reported.

**Table 5:** **Academic resilience scale (personal vignette) item summary statistics by academic self-efficacy (ASE) group**.

**Item**	**Mean (*****SD*****)**		**Mean difference**	**Effect size *d***
***1 = disagree…5 = agree***	**Lower ASE (*n* = 118)**	**Higher ASE (*n* = 106)**		
1. I would not accept the tutors' feedback	0.76	0.69	0.07	0.06
	(1.1)	(1.1)		
2. I would use the feedback to improve my work	4.67	4.92	0.30^*^	0.57
	(0.56)	(0.27)		
3. I would just give up	0.72	0.22	0.50^*^	0.68
	(0.88)	(0.57)		
4. I would use the situation to motivate myself	4.08	4.41	0.33^*^	0.35
	(0.90)	(0.93)		
5. I would change my career plans	0.99	0.58	0.41^*^	0.53
	(0.94)	(0.96)		
6. I would probably get annoyed	2.53	2.48	0.05	0.04
	(1.10)	(1.30)		
7. I would begin to think my chances of success at university were poor	2.05	1.65	0.40^*^	0.37
	(1.12)	(1.10)		
8. I would see the situation as a challenge	3.61	4.19	0.58^*^	0.60
	(0.99)	(0.95)		
9. I would do my best to stop thinking negative thoughts	3.72	4.12	0.40^*^	0.42
	(0.94)	(0.94)		
10. I would see the situation as temporary	3.66	3.98	0.32^*^	0.34
	(0.86)	(1.01)		
11. I would work harder	4.50	4.86	0.36^*^	0.57
	(0.78)	(0.42)		
12. I would probably get depressed	1.87	1.50	0.37^*^	0.30
	(1.14)	(1.30)		
13. I would try to think of new solutions	4.04	4.24	0.37^*^	0.51
	(0.79)	(0.69)		
14. I would be very disappointed	3.23	3.26	0.04	0.03
	(1.05)	(1.15)		
15. I would blame the tutor	0.80	0.56	0.24^*^	0.27
	(0.96)	(0.81)		
16. I would keep trying	4.37	4.80	0.43^*^	0.72
	(0.70)	(0.47)		
17. I would not change my long-term goals and ambitions	3.97	4.38	0.41^*^	0.43
	(0.98)	(0.92)		
18. I would use my past successes to help motivate myself	4.07	4.58	0.51^*^	0.59
	(0.99)	(0.73)		
19. I would begin to think my chances of getting the job I want were poor	1.99	1.24	0.76^*^	0.67
	(1.14)	(1.10)		
20. I would start to monitor and evaluate my achievements and effort	3.86	4.13	0.28^*^	0.48
	(0.94)	(0.94)		
21. I would seek help from my tutors	4.03	4.64	0.61^*^	0.69
	(1.12)	(0.57)		
22. I would give myself encouragement	3.85	4.29	0.45^*^	0.49
	(0.94)	(0.86)		
23. I would stop myself from panicking	3.20	3.76	0.56^*^	0.51
	(1.14)	(1.10)		
24. I would try different ways to study	3.85	4.23	0.38^*^	0.41
	(0.97)	(0.90)		
25. I would set my own goals for achievement	4.00	4.31	0.31^*^	0.38
	(0.82)	(0.81)		
26. I would seek encouragement from my family and friends	3.58	3.77	0.19	0.14
	(1.33)	(1.35)		
27. I would try to think more about my strengths and weaknesses to help me work better	3.92	4.29	0.38^*^	0.44
	(0.86)	(0.82)		
28. I would feel like everything was ruined and was going wrong	1.80	1.22	0.58^*^	0.48
	(1.22)	(1.22)		
29. I would start to self-impose rewards and punishments depending on my performance	2.66	2.90	0.24	0.19
	(1.22)	(1.30)		
30. I would look forward to showing that I can improve my grades	4.07	4.56	0.49^*^	0.58
	(0.94)	(0.73)		

* *p < 0.001, F_(1, 222)_ for univariate tests*.

Table 6 shows ASR-30 (vicarious vignette) mean item scores by ASE group (lower and higher). A One-way MANOVA was performed on these data with ASE group (lower vs. higher) as the independent variable and ASR-30 item scores as the dependent variables. The multivariate effect was non-significant [*F*_(1, 205)_ = 0.659, *p* > 0.05]. Significant univariate effects were only found for items 6, 11, 15 and 24 (*p* < 0.05) and reflect scores indicating significantly higher academic resilience for the higher ASE group, although effect sizes were small or minimal (*d* < 0.2).

**Table 6:** **Academic resilience scale (vicarious vignette) item summary statistics by academic self-efficacy (ASE) group**.

**Item**	**Mean (*****SD*****)**		**Mean difference**	**Effect size *d***
***1 = disagree …5 = agree***	**Lower ASE (*n* = 100)**	**Higher ASE (*n* = 107)**		
1. He should not accept the tutors' feedback	0.74	0.65	0.09	0.08
	(1.11)	(1.14)		
2. He should use the feedback to improve his work	04.72	4.78	0.06	0.08
	(0.70)	(0.79)		
3. He should just give up	0.18	0.16	0.02	0.03
	(0.59)	(0.62)		
4. He should use the situation to motivate himself	4.43	4.53	0.10	0.10
	(0.98)	(0.96)		
5. He should change his career plans	1.01	1.05	0.04	0.04
	(0.94)	(0.99)		
6. He would probably get annoyed	1.25	0.90	0.35^*^	0.33
	(1.17)	(0.92)		
7. He should begin to think his chances of success at university were poor	0.73	0.53	0.20	0.24
	(0.94)	(0.69)		
8. He should see the situation as a challenge	4.24	4.29	0.05	0.06
	(0.78)	(0.77)		
9. He should do his best to stop thinking negative thoughts	4.37	4.46	0.09	0.10
	(0.90)	(0.90)		
10. He should see the situation as temporary	4.01	3.98	0.03	0.03
	(0.94)	(1.17)		
11. He should work harder	4.43	4.67	0.24^*^	0.32
	(0.83)	(0.67)		
12. He would probably get depressed	1.41	1.27	0.14	0.13
	(1.10)	(1.00)		
13. He should try to think of new solutions	4.21	4.35	0.14	0.16
	(0.88)	(0.85)		
14. He would be very disappointed	2.73	2.70	0.03	0.02
	(1.24)	(1.24)		
15. He should blame the tutor	0.71	0.61	0.30^*^	0.34
	(1.05)	(0.67)		
16. He should keep trying	4.73	4.83	0.10	0.15
	(0.69)	(0.61)		
17. He should not change his long-term goals and ambitions	4.30	4.32	0.02	0.02
	(0.91)	(0.90)		
18. He should use his past successes to help motivate himself	4.58	4.63	0.05	0.07
	(0.68)	(0.68)		
19. He should begin to think his chances of getting the job he wants were poor	0.85	0.67	0.08	0.08
	(1.13)	(0.85)		
20. He should start to monitor and evaluate his achievements and effort	4.29	4.42	0.13	0.16
	(0.86)	(0.79)		
21. He should seek help from his tutors	4.75	4.86	0.11	0.20
	(0.61)	(0.50)		
22. He should give himself encouragement	4.56	4.74	0.18	0.27
	(0.70)	(0.65)		
23. He should stop himself from panicking	4.35	4.46	0.11	0.14
	(0.80)	(0.77)		
24. He should try different ways to study	4.44	4.56	0.21^*^	0.16
	(0.80)	(0.66)		
25. He should set his own goals for achievement	4.45	4.54	0.09	0.12
	(0.74)	(0.73)		
26. He should seek encouragement from his family and friends	4.34	4.51	0.17	0.20
	(0.82)	(0.81)		
27. He should try to think more about his strengths and weaknesses to help him work better	4.54	4.64	0.10	0.15
	(0.70)	(0.62)		
28. He should feel like everything was ruined and was going wrong	1.05	0.80	0.25	0.22
	(1.22)	(1.10)		
29. He should start to self-impose rewards and punishments depending on his performance	3.14	3.10	0.04	0.03
	(1.18)	(1.16)		
30. He should look forward to showing that he can improve my grades	4.53	4.64	0.11	0.15
	(0.76)	(0.69)		

* *p < 0.05, F_(1, 205)_ for univariate tests*.

Table 7 shows ASR-30 mean item scores by vignette group. A One-way MANOVA was performed on these data with vignette group (personal vs. vicarious) as the independent variable and ARS-30 item scores as the dependent variables. There was a significant multivariate effect [*F*_(1, 430)_ = 14.929, *p* < 0.001] and significant univariate effects. Significant univariate effects are denoted by “^*^” and “^**^” and reflect scores indicating significantly higher academic resilience for the vicarious group on all items except items 5 and 19 where academic resilience was significantly lower in the vicarious group (with minimal or small effect size) and items 1, 2, 10, 11, 13, 15, and 17, where any differences were non-significant (*p* > 0.05). Effect sizes were large (*d* ≥ 0.8) for one item, medium (*d* ≥ 0.5) for seven items, small (*d* ≥ 0.2) for 12 items, and minimal (*d* < 0.2) for the remaining three items where a significant group difference was reported.

**Table 7:** **Academic resilience scaleitem mean scores by vignette group**.

**Item**	**Mean (*****SD*****)**		**Mean difference**	**Effect size *d***
***1 = disagree …5 = agree***	**Personal vignette (*n* = 224)**	**Vicarious vignette (*n* = 208)**		
1. I would [He should] not accept the tutors' feedback	0.73	0.69	0.04	0.04
	(1.10)	(1.13)		
2. I would [He should] use the feedback to improve my work	4.79	4.75	0.04	0.06
	(0.46)	(0.75)		
3. I would [He should] just give up	0.48	0.17	0.31^**^	0.44
	(0.79)	(0.60)		
4. I would [He should] use the situation to motivate myself	4.23	4.49	0.25^*^	0.28
	(0.93)	(0.96)		
5. I would [He should] change my career plans	0.80	1.03	0.23^*^	0.24
	(0.97)	(0.96)		
6. I [He] would probably get annoyed	2.51	1.07	1.44^**^	1.27
	(1.21)	(1.06)		
7. I would [He should] begin to think my chances of success at university were poor	1.86	0.62	1.24^**^	1.27
	(1.11)	(0.83)		
8. I would [He should] see the situation as a challenge	3.88	4.26	0.38^**^	0.40
	(1.10)	(0.77)		
9. I would [He should] do my best to stop thinking negative thoughts	3.91	4.42	0.51^**^	0.55
	(0.96)	(0.90)		
10. I would [He should] see the situation as temporary	3.81	3.99	0.17	0.18
	(0.94)	(1.07)		
11. I would [He should] work harder	4.67	4.56	0.11	0.16
	(0.66)	(0.76)		
12. I [He] would probably get depressed	1.70	1.33	0.37^**^	0.32
	(1.23)	(1.05)		
13. I would [He should] try to think of new solutions	4.22	4.28	0.07	0.07
	(0.76)	(0.86)		
14. I would [He should] be very disappointed	3.25	2.72	0.53^**^	0.45
	(1.10)	(1.23)		
15. I would [He should] blame the tutor	0.68	0.55	0.13	0.15
	(0.90)	(0.88)		
16. I would [He should] keep trying	4.58	4.78	0.21^**^	0.31
	(0.64)	(0.65)		
17. I would [He should] not change my long-term goals and ambitions	4.16	4.31	0.15	0.16
	(0.97)	(0.90)		
18. I would [He should] use my past successes to help motivate myself	4.31	4.61	0.30^**^	0.37
	(0.91)	(0.68)		
19. I would [He should] begin to think my chances of getting the job I want were poor	0.63	0.81	0.83^**^	0.17
	(1.18)	(0.99)		
20. I would [He should] start to monitor and evaluate my achievements and effort	3.99	4.36	0.37^**^	0.42
	(0.95)	(0.82)		
21. I would [He should] seek help from my tutors	4.32	4.81	0.49^**^	0.63
	(0.95)	(0.56)		
22. I would [He should] give myself encouragement	4.06	4.65	0.60^**^	0.72
	(0.93)	(0.68)		
23. I would [He should] stop myself from panicking	3.47	4.41	0.94^**^	0.97
	(1.13)	(0.78)		
24. I would [He should] try different ways to study	4.03	4.55	0.53^**^	0.61
	(0.95)	(0.73)		
25. I would [He should] set my own goals for achievement	4.15	4.50	0.35^**^	0.45
	(0.83)	(0.74)		
26. I would [He should] seek encouragement from my family and friends	3.67	4.43	0.76^**^	0.69
	(1.34)	(0.81)		
27. I would [He should] try to think more about my strengths and weaknesses to help me work better	4.09	4.59	0.50^**^	0.65
	(0.86)	(0.66)		
28. I would [He should] feel like everything was ruined and was going wrong	1.52	0.92	0.60^**^	0.50
	(1.25)	(1.15)		
29. I would [He should] start to self-impose rewards and punishments depending on my performance	2.77	3.13	0.35^**^	0.30
	(1.26)	(1.17)		
30. I would [He should] look forward to showing that I can improve my grades	4.30	4.58	0.28^**^	0.35
	(0.88)	(0.72)		

* p < 0.01

** *p < 0.001, F_(1, 430)_ for univariate tests*.

**Figure 2:**
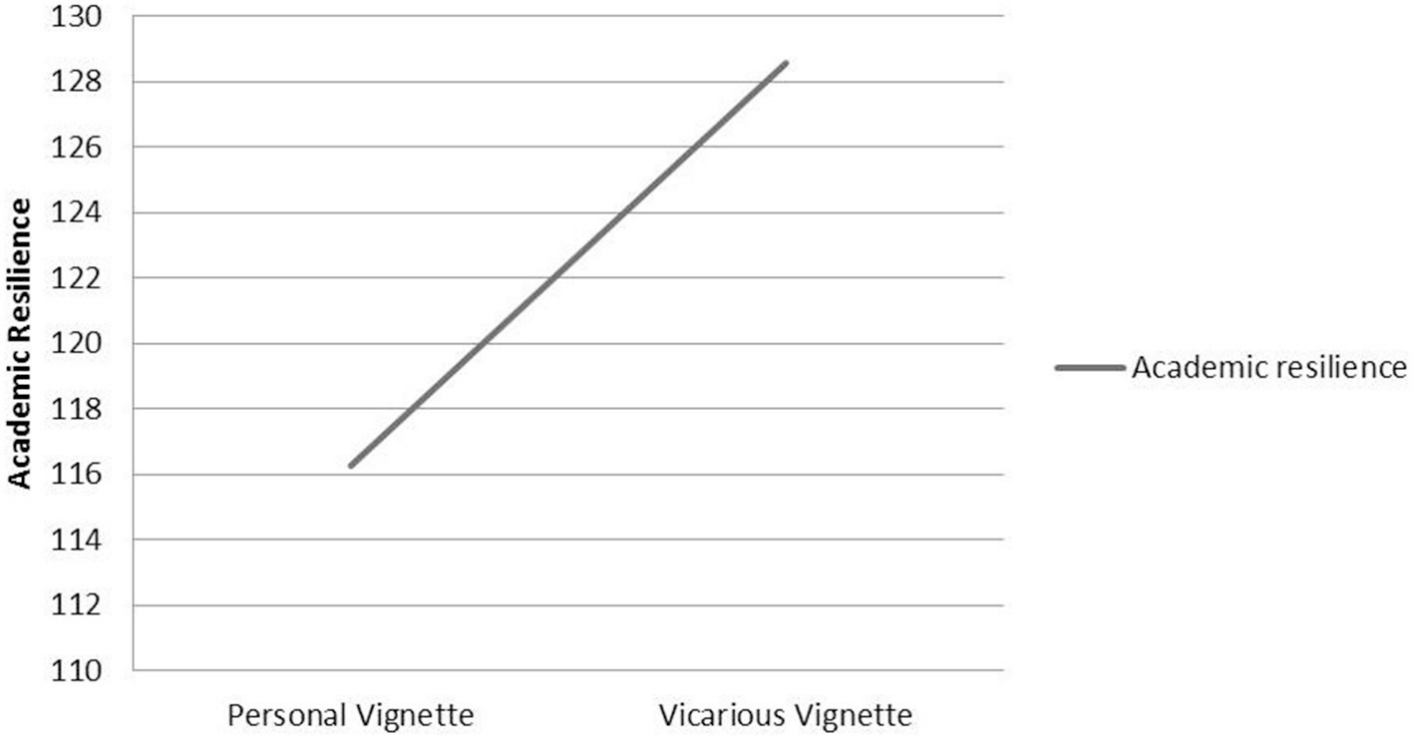
**Mean academic resilience by vignette group**.

Figure 2 shows that while the difference in mean academic resilience scores between the personal and vicarious vignette groups was significant [*t*_(430)_ = 9.908, *p* < 0.001], with a large effect size (*d* = 0.96), there was no significant difference in ASE scores [*t*_(432)_ = 0.356, *p* > 0.05].

### Age, gender, and year of study analysis

Gender and year of study analysis did not reveal any significant differences in academic resilience (*p* > 0.05). Correlational analysis did not reveal any significant association between age and academic resilience (*p* > 0.05), although a small significant correlation between age and ASE [*r*_(429)_ = 1.58, *p* < 0.001] was reported.

## Discussion

Overall results support the hypothesis that ASE is associated with, and a predictor of, academic resilience. Significant positive correlations between ASE and academic resilience were reported for both the personal and vicarious vignettes, although effect size was large for the personal vignette group and small for the vicarious vignette group. Analysis of ASE as a predictor of academic resilience also led to significant results for each of the vignette groups, with the greatest variance in academic resilience (26.2%) accounted for in the personal vignette group compared to only 4.6% in the vicarious group. Although previous studies have reported self-efficacy as an important contributory factor for resilience (Hamill, 2003; Martin and Marsh, 2006), the present study offers additional insight into the context-specific interplay of these constructs. As advocated by Pajares (1996) and by Riley and Masten (2005), Liddle (1994) and Waxman et al. (2003), both self-efficacy and resilience were measured as context-specific constructs and in relation to—it is argued here—an authentic adverse situation and relevant adaptive responses. In both general and context-specific terms, findings support the relevance of self-efficacy beliefs to individual psychological resilience; having positive self-efficacy beliefs is likely to contribute toward increased resilience in students.

Once a relationship between ASE and academic resilience was established, further analysis sought to identify differences between lower and higher self-efficacy students in their specific responses to adversity. As anticipated, higher self-efficacy students reported significantly higher academic resilience for both case vignettes, although a significant interaction effect indicated greater influence of self-efficacy for the personal vignette, where the effect size was large, than for the vicarious vignette, where the effect size was small. The greater influence of self-efficacy on personal resilience is unsurprising in light of Bandura's (1993) account of self-efficacy as a mechanism of *personal* agency that makes causal contributions to *own* functioning. Analysis of responses to individual items on the Academic Resilience Scale-30 (personal vignette) showed that the higher self-efficacy group scored significantly higher on 25 of the 30 items, with small to medium effect sizes reported (see Table 5). This level of analysis highlights specific examples of responses to adversity where self-efficacious students responded in a more adaptive manner, providing a basis to better understand the precise nature of the influence of self-efficacy on resilience and offering a potential basis for interventions promoting resilience. Conversely, the items where there was no significant difference between self-efficacy groups are of little value in differentiating resilient and non-resilient students, at least on the basis of ASE beliefs. Responses to these items could still be adaptive or non-adaptive, conferring resilience or lack of it, but may be determined by individual difference constructs or processes other than self-efficacy. Similar analysis of responses to the vicarious adversity vignette revealed significant differences in only 4 of the 30 items, all with small effect sizes. This further supports the nature of self-efficacy as a mechanism for personal (human) agency and illustrates the limited influence of self-efficacy beliefs on the potential to perform academic advocacy roles, such as peer assisted learning mentors.

Results comparing responses to personal and vicarious vignettes revealed a significant difference and large effect size, with students reporting significantly higher resilience for the vicarious adversity vignette (see Figure 2). This effect was not explained by group differences in self-efficacy. That students advocate more positive adaptive responses to adversity experienced by a peer provides potentially valuable insights for resilience building. In general terms, it supports the value of peer mentoring and peer assisted learning and lessens concerns that negative belief systems might impact negatively on academic advocacy. In fact results suggest that students, including those with lower self-efficacy, are likely to be a positive source of encouragement and resilience for peers who are experiencing challenge and adversity. This is an important finding given continued growth in the implementation, evaluation and reputed benefits of peer assisted learning initiatives (Ginsburg-Block et al., 2006; Smith et al., 2007; Romito, 2014). In more specific terms, results suggest that students are aware of what are and are not adaptive responses and have the potential to exhibit greater personal resilience than they may be currently exhibiting. One aspect of interventions promoting resilience could involve highlighting this difference between personal and vicarious resilience and encouraging students to reflect on their own reasons for advocating greater resilience for their peers and to explore the potential to move toward greater personal adoption of the responses advocated for their peers. Using examples of differences in specific responses, where significant differences in 23 of the 30 items are reported (see Table 7), could be helpful in this respect, enabling students to focus on areas where responses could be more adaptive.

Whilst academic resilience was significantly higher for the vicarious vignette for both lower and higher self-efficacy groups, the difference between personal and vicarious vignettes was greatest for lower self-efficacy students (see Figure 1). One interpretation of this is that lower ASE students have more to gain than students with higher self-efficacy from reflecting on how they respond to adversity experienced by a peer and using this to help promote more adaptive responses to personal adversity.

Consistent with previous studies (Munro and Pooley, 2009; McLafferty et al., 2012), no significant differences in academic resilience according to age, gender, or year of study were observed in the present study. That females were heavily underrepresented in the sample does limit confidence in this particular finding, particularly in light of studies that do report greater academic resilience in female undergraduates (e.g., Allan et al., 2014).

### Limitations

Although the study offers advances in applied academic resilience research and practice, some important limitations need to be considered when interpreting the results and conclusions of the study. Resilience studies commonly operationalize adversity in terms of difficult or unpleasant situations or experiences. It is suggested that the case vignettes developed for the study represent adversity in a relevant and authentic way for the purposes of studying academic adversity. Others—Martin and Marsh (2008, 2009) and Martin (2013) for example—may argue that the vignette is not sufficiently traumatic, stressful or prolonged to adequately represent adversity as it is routinely represented in resilience studies. The ARS-30 is a newly developed measure of academic resilience and although findings do support its reliability and validity, further development work, particularly examining its predictive validity, will add to its integrity as a measure of academic resilience. Comparisons of personal and vicarious resilience were made between subject groups, introducing individual difference error; within-subject comparisons would provide a more robust basis upon which to draw conclusions regarding this aspect of the study. Also, given the differences that emerged between responses to the personal and vicarious case vignettes, those parts of the analysis that combine resilience response data across the vignettes should be treated with caution, focussing instead on analyses presented for the vignettes independently.

### Future directions

Whilst the lack of consensus that exists in terms of conceptualizing and operationalizing resilience (Maclean, 2004; Friedland, 2005) is less pronounced within the narrower field of academic resilience (see Dweck, 2010; Duckworth, 2013; Martin, 2013), it is nonetheless suggested that there are two key areas of development necessary for increased impact of future general and academic resilience research. The first should address how best to capture aspects of resilience in a valid and reliable construct measure or measures. Grotberg (1997) for example summarizes the three aspects of resilience as: “I have” (e.g., trusting and loving relationships, encouragement to be independent); “I am” (e.g., proud of myself, responsible, hopeful); and “I can” (e.g., manage my feelings, solve problems). Similarly, caring relationships, good problem solving and intellectual functioning are identified by Masten and Coatsworth (1998) as factors promoting competency in individuals faced with adversity. The second area of development should continue to address the issue of identifying key factors and constructs associated with resilience. Discussing building resilience in vulnerable and disadvantage children and young people, Maclean (2004) identifies several familiar “qualities” or factors associated with resilience. These include initiative and insight, optimism, intellectual ability, placid temperament, trust, autonomy and decision making, humor, identity, social support, education, attainment, self-esteem and self-efficacy. Maclean goes on to raise the issue of the lack clarity surrounding how practioners can help individuals become more resilient; identifying associated constructs, as Duckworth's (2013) and Dweck's (2006, 2010) have done with their constructs of grit and mindset, will assist the development and implementation of interventions promoting resilience, both in general and academic contexts. Evaluating new interventions is clearly a further avenue for research exploring academic resilience. Other avenues include longitudinal cohort studies examining the predictive value of academic resilience against outcomes including achievement, student satisfaction, retention and wellbeing.

In light of a recent impetus for intrapersonal research in education (Network on Intrapersonal Research in Education, 2015), future studies should consider examining both inter-individual and intra-individual variation in academic resilience. Such studies would reveal the extent to which population data can be generalized to patterns of resilience observed in individual students (and vice-versa), and would be particularly valuable in helping explore process aspects of resilience, as opposed to outcomes measures such as grade point average, in the evaluation of interventions or where adverse situations occur and are time-bound. Windle et al.'s (2011) description of resilience as the process of negotiating, managing and adapting to significant sources of stress or trauma emphasizes the importance of adopting such a process-focused view of resilience.

## Conclusions

The present study sought to identify factors that contribute, in a meaningful way, to academic resilience and to examine how such factors influence specific, and meaningful, responses to academic adversity. Consistent with previous studies (Hamill, 2003; Martin and Marsh, 2006), findings presented support ASE as predictive of academic resilience and go beyond earlier studies in identifying specific examples of responses to academic adversity, where lower and higher self-efficacy students respond in a differentially adaptive manner. As such, it is suggested that self-efficacy training, already shown to be effective in an educational context (Siegle and McCoach, 2007), offers one approach to building academic resilience in students. Illustrating how self-efficacy influences specific responses to adversity, and the propensity to advocate greater resilience for peers facing adversity, should form another—metacognitive—aspect of resilience building for students. As Martin and Marsh (2006) have stated, identifying the specific facets comprising academic resilience will support an enhanced and more targeted approach to interventions aimed at enabling students to cope with the demands of academic life.

### Conflict of interest statement

The author declares that the research was conducted in the absence of any commercial or financial relationships that could be construed as a potential conflict of interest.

## References

[B1] Abiola T. Udofia O. (2011). Psychometric assessment of the Wagnild and Young's resilience scale in Kano, Nigeria. BMC Res. Notes 4:509. 10.1186/1756-0500-4-509 22112503 PMC3261834

[B2] Allan J. F. McKenna J. Dominey S. (2014). Degrees of resilience: profiling psychological resilience and prospective academic achievement in university inductees. Br. J. Guid. Couns. 42, 9–25. 10.1080/03069885.2013.793784

[B3] Alva S. A. (1991). Academic invulnerability among Mexican American students: the importance ofprotective resources and appraisals. Hisp. J. Behav. Sci. 13, 18–34. 10.1177/07399863910131002

[B4] Bandura A. (1993). Perceived self-efficacy in cognitive development and functioning. Educ. Psychol. 28, 117–128. 10.1207/s15326985ep2802_3

[B5] Bandura A. (ed.). (1995). Self-efficacy in Changing Societies. New York, NY: Cambridge University Press.

[B6] Bandura A. (1997). Self-efficacy: The Exercise of Control. New York, NY: Freeman.

[B7] Bandura A. Barbaranelli C. Caprara G. V. Pastorelli C. (2001). Self-efficacy beliefs as shapers of children's aspirations can career trajectories. Child Dev. 72, 187–206. 10.1111/1467-8624.00273 11280478

[B8] Bandura A. Pastorelli C. Barbaranelli C. Caprara G. V. (1999). Self-efficacy pathways to childhood depression. J. Personal. Soc. Psychol. 76, 258–269. 10074708 10.1037//0022-3514.76.2.258

[B9] Bartley M. Schoon M. R. Blane M. (2010). Resilience as an asset for healthy development, in Health Assets in a Global Context, eds Morgan A. Davies M. Ziglio E. (New York, NY: Springer), 101–115.

[B10] Bernard B. (1993). Fostering resiliency in kids. Educ. Leadersh. 51, 44–48.

[B11] Bourne V. (2014). To what extent is mathematical ability predictive of performance in a methodology and statistics course? Can an action research approach be used to understand the relevance of mathematical ability in psychology undergraduates. Psychol. Teach. Rev. 20, 14–27.

[B12] Cassidy S. (2012). Exploring individual differences as determining factors in student academic achievement in higher education. Stud. High. Educ. 37, 793–810. 10.1080/03075079.2010.545948

[B13] Cassidy S. Eachus P. (2002). The development of the General academic self- efficacy (GASE) scale, in Paper presented at the British Psychological Society Annual Conference (Blackpool).

[B14] Cattell R. B. (1966). The scree test for the number of factors. Multivariate Behav. Res. 1, 245–276. 10.1207/s15327906mbr0102_10 26828106

[B15] Cohen J. (1988). Statistical Power Analysis for the Behavioral Sciences, 2nd Edn. Hillsdale, NJ: Lawrence Earlbaum Associates.

[B16] Duckworth A. (2013). The key to success? Grit. Ted Talks Education: Interactive transcript. Available online at: http://www.ted.com/talks/angela_lee_duckworth_the_key_to_success_grit?language=en

[B17] Duckworth A. L. Peterson C. Matthews M. D. Kelly D. R. (2007). Grit: perseverance and passion for long-term goals. J. Pers. Soc. Psychol. 9, 1087–1101. 10.1037/0022-3514.92.6.1087 17547490

[B18] Duckworth A. L. Quinn P. D. (2009). Development and validation of the Short Grit Scale (Grit-S). J. Pers. Assess. 91, 166–174. 10.1080/00223890802634290 19205937

[B19] Dweck C. S. (2006). Mindset: The New Psychology of Success. New York, NY: Random House.

[B20] Dweck C. S. (2010). Mind-sets and equitable education. Principal Leadersh. 10, 26–29.

[B21] Eachus P. (1993). Development of the health student self-efficacy scale. Percept. Mot. Skills 77:670. 10.2466/pms.1993.77.2.670 8247691

[B22] Farrington C. A. Roderick M. Allensworth E. A. Nagaoka J. Johnson D. W. Keyes T. S. (2012). Teaching Adolescents to Become Learners: The Role of Noncognitive Factors in Academic Performance—A Critical Literature Review. Chicago: Consortium on Chicago School Research.

[B23] Farrington D. P. Loeber R. (2000). Some benefits of dichotomization in psychiatric and criminological research. Crim. Behav. Ment. Health 10, 100–122. 10.1002/cbm.349

[B24] Field A. (2014). Discovering Statistics using IBM SPSS Statistics, 4th Edn London: Sage.

[B25] Friedland N. (2005). Introduction – The ‘elusive’ concept of social resilience, in The Concept of Social Resilience, eds Friedland N. Arian A. Kirschnbaum A. Karin A. Fleischer N. (Haifa: The Technion. Samuel Neaman Institute, 7–10.

[B26] Garmezy N. Masten A. S. (1991). The protective role of competence indicators in children at risk, in Life-span Developmental Psychology: Perspectives on Stress and Coping, eds Cummings E. M. Greene A. L. Karraker K. H. (Hillsdale, NJ: Erlbaum), 151–174.

[B27] Gilligan R. (2001). Promoting Resilience: A Resource Guide on Working with Children in the Care System. London: British Agencies for Adoption and Fostering.

[B28] Ginsburg-Block M. D. Rohrbeck C. A. Fantuzzo J. W. (2006). A meta-analytic review of social, self-concept, and behavioral outcomes of peer-assisted learning. J. Educ. Psychol. 98, 732–749. 10.1037/0022-0663.98.4.732

[B29] Grotberg E. (1997). The international resilience project: findings from the research and effectiveness interventions, in Psychology and Education in the 21st Century: Proceedings of the 54th Annual Convention, ed Bain B. (Edmonton: ICP Press).

[B30] Hamill S. K. (2003). Resilience and self-efficacy: the importance of efficacy beliefs and coping mechanisms in resilient adolescents. Colgate Univ. J. Sci. 35, 115–146.

[B31] Hatcher L. (1994). A Step-by-Step Approach to using the SAS System for Factor Analysis and Structural Equation Modeling. Cary, NC: SAS Institute Inc.

[B32] Irwin J. R. McClelland G. H. (2003). Negative consequences of dichotomizing continuous predictor variables. J. Mark. Res. 40, 366–371. 10.1509/jmkr.40.3.366.19237

[B33] Kaiser H. F. (1960). The application of electronic computers to factor analysis. Educ. Psychol. Meas. 20, 141–151. 10.1177/001316446002000116

[B34] Kluger A. N. DeNisi A. (1996). The effects of feedback interventions on performance: a historical review, a meta-analysis, and a preliminary feedback intervention theory. Psychol. Bull. 119, 254–284.

[B35] Liddle H. A. (1994). Contextualizing resiliency, in Educational Resilience in Inner-city America: Challenges and Prospects, eds Wang M. C. Gordon E. W. (Hillsdale, NJ: Erlbaum), 167–177.

[B36] MacCallum R. C. Zhang S. Preacher K. J. Rucker D. D. (2002). On the practice of dichotomization of quantitative variables. Psychol. Methods 7, 19–40. 10.1037/1082-989X.7.1.19 11928888

[B37] Martin A. Marsh H. (2006). Academic resilience and its psychological and educational correlates: a construct validity approach. Psychol. Sch. 43, 267–281. 10.1002/pits.20149

[B38] Martin A. J. (2013). Academic buoyancy and academic resilience: exploring ‘everyday’ and ‘classic’ resilience in the face of academic adversity. Sch. Psychol. Int. 34, 488–500. 10.1177/0143034312472759

[B39] Martin A. J. Marsh H. W. (2008). Academic buoyancy: Towards an understanding of students' everyday academic resilience. J. Sch. Psychol. 46, 53–83. 10.1016/j.jsp.2007.01.002 19083351

[B40] Martin A. J. Marsh H. W. (2009). Academic resilience and academic buoyancy: multidimensional and hierarchical conceptual framing of causes, correlates and cognate constructs. Oxf. Rev. Educ. 35, 353–370. 10.1080/03054980902934639

[B41] Masten A. S. Coatsworth J. D. (1998). The development of competence in favorable and unfavorable environments. Am. Psychol. 53, 205–220. 9491748 10.1037//0003-066x.53.2.205

[B42] McLafferty M. Mallet J. McCauley V. (2012). Coping at university: the role of resilience, emotional intelligence, age and gender. J. Quant. Psychol. Res. 1, 1–6.

[B43] Maclean K. (2004). Resilience: what it is and how children and young people can be helped to develop it, in Online Journal of the International Child and Youth Care Network, Vol. 62 Available online at: http://www.cyc-net.org/cyc-online/cycol-0304-resilience.html

[B44] McMillan J. H. Reed D. F. (1994). At-risk students and resiliency: factors Contributing to academic success. Clearing House 67, 137–140.

[B45] Munro B. Pooley J. A. (2009). Differences in resilience and university adjustment between school leaver and mature entry university students. Aust. Commun. Psychol. 21, 50–61.

[B46] Network on Intrapersonal Research in Education (2015). Learning Processes: Theoretical and Conceptual Issues. Oxford: Department of Education, University of Oxford.

[B47] Nunnally J. C. Bernstein I. H. (1994). Psychometric Theory, 3rd Edn. New York, NY: McGraw-Hill.

[B48] Ozer E. Bandura A. (1990). Mechanisms governing empowerment effects: a self-efficacy analysis. J. Pers. Soc. Psychol. 58, 472–486. 2324938 10.1037//0022-3514.58.3.472

[B49] Pajares F. (1996). Self-efficacy beliefs in academic settings. Rev. Educ. Res. 66, 543–578. 10.3102/00346543066004543

[B50] Parkinson J. (2008). Review of Scales of Positive Mental Health Validated for use with Adults in the UK: Technical Report. Glasgow: NHS Health Scotland.

[B51] Perkins-Gough D. (2013). The significance of grit: a conversation with Angele Lee Duckworth. Educ. Leadersh. 71, 14–20.

[B52] Pooley J. Cohen L. (2010). Resilience: a definition in context. Aust. Commun. Psychol. 22, 30–37.

[B53] Riley J. R. Masten A. S. (2005). Resilience in context, in Resilience in Children, Families, and Communities: Linking Context to Practice and Policy, eds Peters R. D. Leadbeater B. McMahon R. (New York, NY: Kluwer Academic/Plenum), 13–25.

[B54] Romito A. (2014). Peer Assisted Learning, in The Essential Handbook for GP Training and Education, ed Mehay R. Available online at: essentialgptrainingbook.com

[B55] Schmitt N. Stults D. M. (1985). Factors defined by negatively keyed items: the results of careless respondents? Appl. Psychol. Meas. 9, 367–373.

[B56] Siegle D. McCoach D. B. (2007). Increasing student mathematics self-efficacy through teacher training. J. Adv. Acad. 18, 278–312.

[B57] Smith J. May S. Burke L. (2007). Peer assisted learning: a case study into the value to student mentors and mentees. Pract. Evid. Sch. Teach. Learn. High. Educ. 2, 80–109.

[B58] Snipes J. Fancsali C. Stoker G. (2012). Student Academic Mindset Interventions: A Review of the Current Landscape. San Francisco: Stupski Foundation Available online at: http://www.impaqint.com/files/4-content/1-6-publications/1-6-2-project-reports/impaq%20student%20academic%20mindset%20interventions%20report%20august%202012.pdf

[B59] Spector P. E. Van Katwyk P. T. Brannick M. T. Chen P. Y. (1997). When two factors don't reflect two constructs: how item characteristics can produce artifactual factors. J. Manage. 23, 659–678. 10.1016/j.jagp.2014.08.003 25214029 PMC4326597

[B60] Stevens J. P. (2002). Applied Multivariate Statistics for the Social Sciences, 4th Edn. Hillsdale, NJ: Erlbaum.

[B61] Wagnild G. M. (2009). The Resilience Scale User's Guide for the US English version of the Resilience Scale and the 14-Item Resilience Scale (RS-14). Worden, MT: The Resilience Centre.

[B62] Wang M. C. Haertel G. D. Walberg H. J. (1994). Educational resilience in inner cities, in Educational Resilience in Inner-city America: Challenges and Prospects, eds Wang M. C. Gordon E. W. (Hillsdale, NJ: Erlbaum), 45–72.

[B63] Waxman H. C. Gray J. P. Padron Y. N. (2003). Review of Research on Educational Resilience: Research Report. Washington, DC: Institute of Education Sciences.

[B64] Windle G. Bennett K. Noyes J. (2011). A methodological review of resilience measurement scales. Health Qual. Life Outcomes 9:8. 10.1186/1477-7525-9-8 21294858 PMC3042897

[B65] World Health Organization (WHO) (2005). WHO Mental Health Declaration for Europe: Facing the Challenges, Building the Solutions. Denmark: World Health Organization Available online at: http://www.euro.who.int/__data/assets/pdf_file/0008/96452/E87301.pdf

[B66] Zautra A. J. (2009). Resilience: one part recovery, two parts sustainability. J. Pers. 77, 1935–1943. 10.1111/j.1467-6494.2009.00605.x 19807859

